# Versatile High‐Sensitivity EPR Using Superconducting Spiral Microresonators

**DOI:** 10.1002/smtd.202501451

**Published:** 2025-11-12

**Authors:** Gediminas Usevičius, Mantas Šimėnas, Blaise L. Geoghegan, Oscar W. Kennedy, Ignas Pocius, Patrick Hogan, Ana Villanueva Ruiz de Temino, Jean‐Baptiste Verstraete, Paulina Verbaitytė, Angeliki Chatziathanasiou, G. Antilen Jacob, Mindaugas Kamarauskas, Marius Treideris, Paulius Gečys, Joseph Alexander, Vidmantas Kalendra, Jūras Banys, Maxie M. Roessler, John J.L. Morton

**Affiliations:** ^1^ Faculty of Physics Vilnius University Sauletekio 3 LT‐10257 Vilnius Lithuania; ^2^ Department of Chemistry and Centre for Pulse EPR Spectroscopy (PEPR), Imperial College Molecular Sciences Research Hub London W12 0BZ UK; ^3^ London Centre for Nanotechnology University College London London WC1H 0AH UK; ^4^ Center for Physical Sciences and Technology Sauletekio 3 Vilnius LT‐10257 Lithuania; ^5^ Dept. of Electronic & Electrical Engineering University College London London WC1E 7JE UK

**Keywords:** DEER, EPR (Electron paramagnetic resonance), microresonator, sensitivity

## Abstract

Electron paramagnetic resonance (EPR) spectroscopy is a broadly used technique to study paramagnetic centers in diverse fields ranging from biology to quantum technologies. The availability of well‐established commercial instrumentation, including features such as rapid sample exchange, has been a key enabler for EPR to be applied widely across disciplines. Here, a three‐order‐of‐magnitude increase is presented in the spin number sensitivity of the commonly used X‐band pulsed EPR while retaining full compatibility with conventional instrumentation and typical sample conditions. This approach employs planar spiral‐shaped microresonators with 7 nL mode volumes fabricated from yttrium barium copper oxide (YBCO) high‐temperature superconductor. A wide range of microwave coupling is achieved by a single microresonator inside a conventional EPR tube, loaded into an EPR cavity. The performance of the spiral microresonators is demonstrated through a suite of pulsed EPR experiments on standard samples, including dipolar and hyperfine spectroscopies. By placing a sample within a microfluidic microstructure fabricated to match the mode profile of the microresonator, a high‐fidelity spin control is obtained with a spin‐number sensitivity of 10^7^ spins/G/$\sqrt{\text{Hz}}$. The approach significantly advances the applicability of superconducting microresonators as versatile and readily applicable tools for high sensitivity EPR.

## Graphical abstract

A significant sensitivity enhancement in conventional X‐band pulsed EPR is achieved using planar spiral microresonators fabricated from high‐temperature superconducting YBCO. Fully compatible with standard instrumentation, the approach enables high‐fidelity spin control under typical sample conditions. The demonstrated performance opens new possibilities for superconducting microresonators as versatile tools in high‐sensitivity EPR spectroscopy.

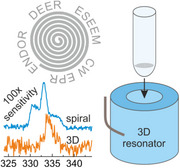

## 1 Introduction

Electron paramagnetic resonance (EPR) is a powerful spectroscopic technique with applications covering structural biology,^[^ ^1^, ^2^, ^3^, ^4^, ^5^ ^]^ spin‐based quantum technologies,^[^ ^6^, ^7^, ^8^, ^9^ ^]^ solid‐state physics,^[^ ^10^, ^11^ ^]^ catalysis,^[^ ^12^, ^13^, ^14^ ^]^ and chemical sciences.^[^ ^15^ ^]^ Despite its versatility, the relatively low sensitivity of conventional EPR poses a significant challenge for studying volume‐limited systems, as standard EPR resonators typically require samples of 10–100 microliter volumes to achieve detectable spin signals.^[^ ^16^ ^]^ To overcome these limitations, considerable effort has been directed toward the development of planar microwave microresonators of various designs for both continuous‐wave (CW)^[^ ^17^, ^18^, ^19^, ^20^, ^21^ ^]^ and pulsed EPR.^[^ ^22^, ^23^, ^24^, ^25^, ^26^, ^27^, ^28^, ^29^, ^30^, ^31^, ^32^ ^]^ Such microresonators feature dramatically reduced mode volumes compared to conventional 3D (e.g., dielectric or loop‐gap) microwave resonators, offering enhanced sensitivity for small‐scale systems.^[^ ^33^, ^34^ ^]^

By fabricating the microresonators from superconducting materials, key dimensions such as inductor width can be reduced resulting in detectable (mode) volumes as small as a few femtoliters, while maintaining a high resonator quality factor (Q‐factor).^[^ ^29^ ^]^ Furthermore, by operating at temperatures of a few milliKelvin, it is possible to benefit from complete spin polarisation and (if quantum‐limited amplifiers are used for detection)^[^ ^25^ ^]^ suppression of noise levels to the vacuum limit. Such techniques have enabled pulsed EPR with spin‐number sensitivity of 12 spins/$\sqrt{\mathrm{Hz}}$ ^[^ ^29^ ^]^ or even at the single spin limit using single‐microwave photon detectors.^[^ ^35^ ^]^ However, milliKelvin temperatures are not (yet) readily accessible in conventional EPR laboratories, and many applications require EPR spectroscopy over a much broader range of temperatures. Furthermore, microresonators exhibit poor spin‐concentration sensitivity compared to 3D cavities, while the use of conventional superconductors necessitates precise alignment of the external magnetic field,^[^ ^36^ ^]^ which further limits their applicability for EPR spectroscopy.

Recent studies have shown that high‐temperature superconductors such as yttrium barium copper oxide (YBCO) can be used to make microresonators with a broad range of operating temperatures and reduced sensitivity to out‐of‐plane magnetic field.^[^ ^30^, ^37^, ^38^ ^]^ YBCO microresonators have been coherently coupled to molecular spin ensembles^[^ ^38^, ^39^, ^40^ ^]^ leading to a pulsed EPR study of organic radicals,^[^ ^32^ ^]^ while YBCO microresonators with sub‐nL mode volumes have been used to measure phosphorus donor spins in isotopically‐enriched ^28^Si.^[^ ^30^ ^]^ However, as these demonstrations aimed to achieve maximum sensitivity for particular samples, they employed specialized probeheads and lacked simple methods to adjust the microwave coupling, significantly limiting their use in wider EPR applications. In summary, substantial practical barriers continue to restrict microresonators to niche EPR applications, despite significant efforts put into their design and their immense potential as highly sensitive tools for EPR of volume‐limited systems.

Here, we introduce a YBCO microresonator design along with a simple approach to microresonator coupling via a standard 3D EPR resonator which together offer the advantages of high spin number sensitivity with the ability to be readily deployed within any typical EPR laboratory. The microresonator features a spiral geometry designed for X‐band (9.5 GHz) EPR with a mode volume of about 7 nL, lying at an intermediate point between that of the 3D EPR cavities (10–100 µL) and the smallest microresonators (≈10 fL). At such volumes, our resonator maintains an acceptable spin‐concentration sensitivity, while significantly enhancing spin‐number sensitivity (down to ≈10^7^ spins/G/$\sqrt{\text{Hz}}$). We demonstrate the performance of our design through a suite of pulsed EPR experiments, such as electron spin echo envelope modulation (ESEEM), electron‐nuclear double resonance (ENDOR), and double electron‐electron resonance (DEER) spectroscopy on various samples, including biological systems. The spin‐number sensitivity of our microresonators exceeds that of 3D resonators by three orders of magnitude, offering a versatile and highly sensitive solution for EPR studies of volume‐limited samples across different fields of EPR spectroscopy. The advancements presented in this work pave the way for microresonators to transition from niche applications into mainstream EPR spectroscopy.

## 2 Results

### 2.1 Microresonator Design and Characterization

**Figure 1:**
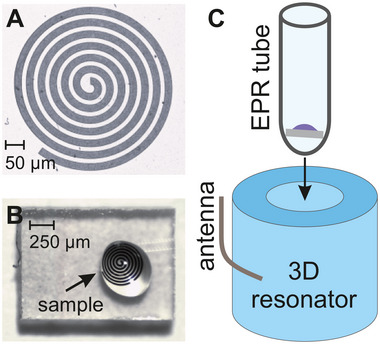
Design and loading of the spiral microresonator. A) Fabricated planar YBCO spiral microwave microresonator on a sapphire substrate. (B) Individual microresonator with an aqueous droplet containing nitroxide radicals. (**C**) The microresonator sits approximately horizontally at the bottom of an EPR tube, which is then loaded into a standard 3D EPR resonator facilitating coupling to a microwave antenna.

Our planar YBCO microresonators feature a spiral distributed‐element design (see **Figure** **1**A), which is expected to provide a more homogeneous microwave magnetic field *B*_1_ distribution in the mode volume compared to a straight inductor geometry.^[^ ^41^ ^]^ The width of the spiral winding is 20 µm with an interwinding spacing of 10 µm. The resonance frequency of the spiral microresonator is determined by its total length with the typical value of 4.5 mm for X‐band. The diameter and impedance of the spiral resonating at 9.5 GHz microwave frequency are 450 µm and 426 Ω, respectively (see Section S1, Supporting Information). The size of the microresonator is comparable to that of a typical minimal liquid droplet sample that we were able to load onto the spiral using a pipette (see Figure 1B). The microwave coupling of the microresonator is achieved by inserting it into a Bruker MD5 sapphire ring dielectric resonator using a conventional X‐band EPR tube (Figure 1C). This approach ensures full compatibility of our microresonators with the current state‐of‐the‐art commercial EPR spectrometers.

**Figure 2:**
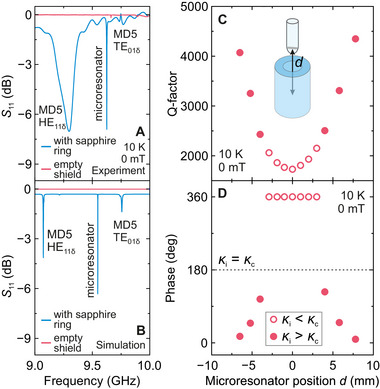
Tunable microwave coupling of the spiral microresonator. A) Experimental and B) simulated *S*_11_ VNA traces of the spiral microresonator situated within a typical Bruker MD5 resonator assembly. If the sapphire ring is removed from the assembly, the microresonator mode is no longer visible. The traces obtained with the MD5 resonator are downshifted for clarity. C) Q‐factor and D) phase of the spiral microresonator as a function of its distance *d* from the center of the Bruker MD5 cavity. The open and filled points correspond to the overcoupled (κ_*c*_ > κ_*i*_) and undercoupled (κ_*c*_ < κ_*i*_) microresonator mode, respectively. The dashed line marks the critical coupling (κ_*c*_ = κ_*i*_) case. Measurements performed at 10 K and 0 mT.

Prior to running the EPR experiments, we studied the resonant and coupling properties of the spiral microresonators using the finite element CST simulations (see Figures S1‐S3, Supporting Information) followed by a thorough experimental characterization of over 120 microresonators using vector network analyzer (VNA) measurements (Figure S3, Supporting Information). The *S*_11_ parameter measured using a VNA of a single spiral inserted into the Bruker MD5 resonator is presented in **Figure** **2**A showing a sharp resonance at 9.6 GHz. In addition, two modes (HE_11δ_ and TE_01δ_) of the sapphire ring can be observed at 9.3 and 9.75 GHz. To confirm that the coupling occurs via the 3D resonator, we repeated the experiment after removing the sapphire ring from the Bruker MD5 resonator assembly. In this configuration, the spiral microresonator showed negligible mode prominence (depth) (Figure 2A) clearly demonstrating that the microwave field generated by the 3D cavity strongly enhances the coupling. Figure 2B shows the CST simulations for the same configurations reproducing analogous behavior of the microresonator coupling (simulation geometry presented in Figure S1). We attribute the coupling mechanism primarily to the inductive coupling mediated by the microwave magnetic field, which is maximal in the center of the sapphire ring where the spiral is located, while the electric field component is nearly absent at this point.^[^ ^42^ ^]^ Such a resonator coupling via the intermediate field of a cavity has previously been implemented for 3D dielectric resonator systems.^[^ ^43^, ^44^, ^45^ ^]^ Note that we also observed overcoupling of the spiral with a Bruker MS3 split‐ring resonator indicating that this effect is not specific to the MD5 geometry.

The advantage of our approach is the ability to adjust the microwave coupling by changing the position and orientation of the spiral microresonator within the sapphire ring resonator. This tunability allows access to different coupling regimes relevant for EPR experiments. At the center of the ring, the microresonator has the lowest Q‐factor, while the phase response has a 360° shift indicating the overcoupled case (see Figure 2C,D; and S4), where the microwave coupling rate to the transmission line κ_*c*_ is higher than the intrinsic loss rate κ_*i*_ of the microresonator. As the spiral is moved away from the center of the sapphire ring, the coupling gradually changes from overcoupled to undercoupled (κ_*c*_ < κ_*i*_) via the critical coupling (κ_*c*_ = κ_*i*_) regime. We reproduced these experimental findings using the CST simulations presented in Figure S5. Note that in most cases the spiral naturally rests approximately horizontally in the EPR tube allowing the overcoupled regime to be easily achieved.

**Figure 3:**
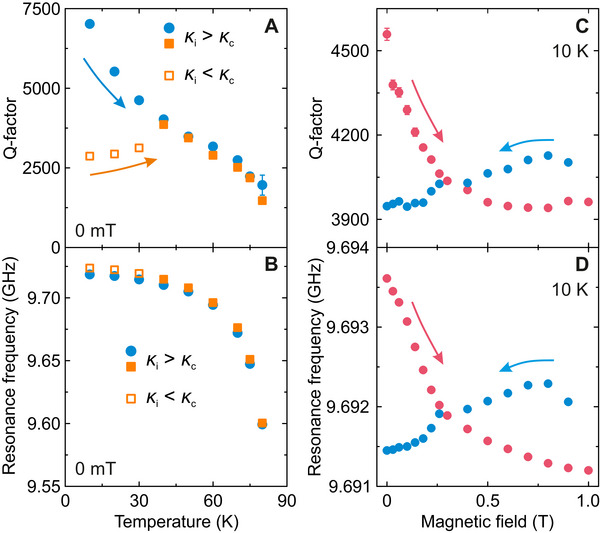
Temperature and magnetic field resilience of the spiral microresonator. A,B) Temperature and C,D) magnetic field dependence of the A,C) Q‐factor and B,D) resonance frequency of the same microresonator. In (A,B), two temperature dependent measurements were performed on heating starting from the overcoupled (κ_*c*_ > κ_*i*_, empty squares) and undercoupled (κ_*c*_ < κ_*i*_, filled circles) regimes of the same microresonator at 10 K. In (C,D), the magnetic field was first increased (red) and then decreased (blue).

In addition to tunable coupling, performance of the microresonator under variable temperatures, magnetic fields and microwave powers are of key importance to EPR applications. The temperature dependences of the microresonator Q‐factor and resonance frequency are presented in **Figure** **3**A,B revealing that the YBCO spiral resonates up to 80 K before losing its superconducting properties, offering a wide operating temperature range sufficient for many pulsed EPR experiments. The resonance frequency exhibits a relatively weak shift with temperature, while the Q‐factor may exhibit more pronounced changes depending on the microresonator coupling. Figure 3A presents two different heating runs starting from the deliberately selected undercoupled and overcoupled regimes of the same spiral. In the former case, the Q‐factor gradually decreases from about 7500 at 10 K to 2500 at 80 K, due to the increase of the intrinsic microwave losses κ_*i*_ of the microresonator. In contrast, starting from the overcoupled case (κ_*c*_ > κ_*i*_), which is more common in pulsed EPR, the Q‐factor starts to decrease only below about 50 K, as the intrinsic losses start to dominate. The initial increase of the Q‐factor below 50 K results from a gradual decrease in κ_*c*_ due to the temperature‐induced shift of the microresonator frequency away from the mode of the 3D cavity (Figure 3B).

The magnetic field dependence of the Q‐factor and resonance frequency of the spiral microresonator is presented in Figure 3C,D. The observed changes of the microresonator Q‐factor (≈15% sweeping up from 0 to 1 T, and ≈5% sweeping back down) as well as in the resonance frequency (<3 MHz between 0 and 1 T) are within bounds acceptable for most EPR applications. The observed shifts are also significantly smaller compared to microresonators fabricated from conventional low‐temperature superconductors (e.g., NbN),^[^ ^36^ ^]^ which must be carefully aligned to prevent out‐of‐plane field components from disrupting the superconducting state. In our case, the YBCO‐based spiral microresonators tolerate much higher out‐of‐plane fields allowing for imperfect horizontal placement within the EPR tube. The hysteretic field response of both Q‐factor and resonator frequency is caused by pinning of magnetic field flux quanta in the superconductor, as observed (with much larger variations) with Nb‐based resonators.^[^ ^46^ ^]^ This effect can be partially mitigated by positioning the spiral as close to the horizontal orientation as possible.

Superconducting resonators are also known to exhibit non‐linear behavior at high microwave powers,^[^ ^47^ ^]^ which we studied using VNA experiments (see Figure S6, Supporting Information). Our YBCO spirals sustain relatively high continuous wave (CW) powers of about 0.1–1 mW, beyond which they start to exhibit pronounced non‐linearity. Such power levels are compatible with the majority of CW EPR experiments at low temperature. Note that during pulsed EPR experiments described further below, we have not detected any effects of the resonance shifting despite the use of much higher instantaneous pulse powers (tens and hundreds of mW). However, depending on the fabrication quality, we observed that spirals containing visible defects disintegrated at very strong pulse powers of about 100 W.

**Figure 4:**
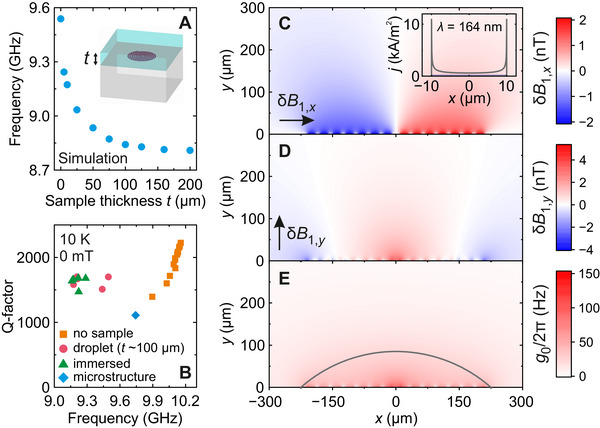
Sample effect and mode volume of the spiral microresonator. A) Finite element CST simulation of the microresonator resonance frequency versus sample (frozen aqueous solution) thickness (see inset). B) Distribution of the Q‐factor and resonance frequency of a selected spiral microresonator for different nitroxide sample geometries (droplet of about 100 µm thickness, full immersion). Multiple sample loadings and microresonator insertions were used to obtain the presented dataset. Spatial distribution of vacuum magnetic field fluctuations (C) δ*B*_1, *x*_ and (D) δ*B*_1, *y*_ of a spiral microresonator corresponding to the current vacuum fluctuations at 10 K (inset shows the current profile) as obtained from finite element COMSOL simulations. E) Spatial distribution of the calculated spin‐resonator coupling strength *g*_0_ for spin species with *g*‐factor of two. The curve indicates cross section of a spherical cap representing the microresonator mode volume.

Subsequently, we investigated the impact of the sample on the microresonator, as its dielectric permittivity is expected to significantly downshift the resonance frequency. We used CST simulations to predict sample‐dependent frequency shifts associated with a frozen aqueous film of varying thickness over the spiral (**Figure** **4**A). We observed a gradual decrease in the resonance frequency with a total frequency shift of about 700 MHz. For thicknesses greater than 100 µm, the frequency shift becomes marginal allowing us to roughly infer the spatial extension of the dominant microwave field generated by the spiral microresonator. Our experimental results from a microresonator with a (subsequently) frozen aqueous droplet (thickness of about 100 µm) placed on top showed a frequency shift of approximately 800 MHz compared to the bare resonator (Figure 4B) in agreement with our CST simulations (Figure 4A). A similar frequency shift was also observed, when fully immersing the spiral into the sample (Figure 4B) indicating that the microresonator mode volume is fully covered with the sample droplet. Note that repeated insertions of the same microresonator into the Bruker MD5 cavity lead to some variations in the measured Q‐factor and frequency, which we attribute to differences in orientation of the spiral within the EPR tube. For bare microresonator, the distribution of the resonance frequency spans about 200 MHz, while the Q‐factor varies by about 50% (Figure 4B). The observed gradual change in Q‐factor arises from a variation in coupling, as the frequency of the spiral approaches the 3D cavity mode. If designing microresonators for specific samples ‐ for example to address transition frequencies of interest ‐ such dielectric frequency shifts must be taken into account.

Having established the impact of the sample on the microresonator properties, we turn to the ability of the spiral to effectively drive EPR transitions of the sample. One of the key challenges of microresonator EPR is managing the large inhomogeneity in the microwave magnetic field $B_{1}\left(r\right)$ which arises as a function of displacement r from the center of the microresonator. This variation can be seen in further finite element simulations (now performed using COMSOL) which account for the distribution of the superconducting current within the spiral winding under a given microwave excitation power (see Section S1, Supporting Information and Experimental Section for more details). The resulting in‐plane (*B*_1, *x*_) and out‐of‐plane (*B*_1, *y*_) field components, shown in Figure 4C,D, illustrate the variation in the strength and orientation of the microwave magnetic field. Here, the values of *B*_1_ have been scaled to give the spatial distribution of the vacuum microwave magnetic field fluctuations $\delta B_{1}\left(r\right)$, which, we shall see below, can be used to quantitatively predict spin‐resonator coupling.^[^ ^25^, ^33^ ^]^ The simulations show that *B*_1, *x*_ dominates above the spiral tracks and is relatively homogeneous apart from the first ≈5μm above the film, while the *B*_1, *y*_ component is maximal in the center. The microwave electric field is predominantly confined within the gaps separating the spiral windings.

We use the calculated $B_{1}\left(r\right)$ distribution to extract the mode volume of the spiral microresonator defined as the volume containing half of the microwave field energy. Based on the shape of the field distribution, we assumed a spherical cap geometry for the mode volume with a base diameter equal to that of the spiral. In this case, the calculated mode volume is 7 nL, and its profile is given in Figure 4E. This mode volume is about four orders of magnitude smaller compared to conventional X‐band 3D resonators providing significant opportunities for spiral microresonators in EPR of minute samples.

In addition to providing a measure of the inhomogeneity of the *B*_1_ field, the calculated spatial distribution of the vacuum microwave magnetic field fluctuations $\delta B_{1}\left(r\right)$ can be used to give quantitative predictions of the single‐spin‐resonator coupling expressed as $g_{0}\left(r\right)=\gamma_{e}\delta B_{1}\left(r\right)$, where γ_*e*_ is the electron gyromagnetic ratio.^[^ ^25^, ^33^ ^]^ The parameter *g*_0_ defines the interaction between a single spin and the resonator, and it plays a major role in determining both the EPR signal intensity and the pulse rotation angle, as outlined in Experimental Section. Assuming an isotropic electron spin with g‐factor *g* = 2, the obtained magnitude of *g*_0_/2π for the spiral microresonator ranges from approximately 150 Hz for spins closest to the spiral gradually decreasing to nearly zero (<4 Hz) at distances beyond a few hundred micrometers (see Figure 4E; Figure S7, Supporting Information). The average value of *g*_0_/2π within the microresonator mode volume is approximately 33 Hz, which is similar in magnitude to some single‐inductor microresonators.^[^ ^25^, ^48^ ^]^

### 2.2 Pulsed EPR and Microresonator Sensitivity

Having established the coupling characteristics, sample influence, and the field and temperature resilience of the spiral microresonators, we benchmark their performance in EPR spectroscopy by conducting pulsed EPR experiments of a 0.1 mM TEMPO droplet sample (see Experimental Section for sample details). Using 5 mW of microwave power delivered at the antenna, we achieved a π‐pulse duration of 40 ns. This allowed us to determine the power‐field conversion factor, defined as $C=B_{1}/\sqrt{P}$, where *P* is microwave power. For a spiral microresonator with *Q* = 1900, we found *C* = 35 G/$\sqrt{W}$, while that of 3D resonators is of the order of 1 G/$\sqrt{W}$. Similar values of *C* have been observed in previously reported microresonator designs.^[^ ^18^, ^20^, ^21^, ^22^, ^30^ ^]^ Such lower microwave powers avoid the need for high‐power traveling‐wave tube (TWT) amplifiers and make it easier to integrate switched cryogenic low noise amplifiers (LNAs)^[^ ^49^ ^]^ into the probehead for higher sensitivity.

**Figure 5:**
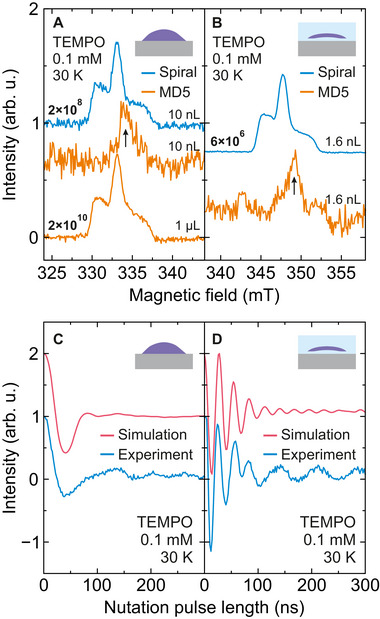
Spin sensitivity and Rabi nutation. A,B) EDFS spectra of a 0.1 mM TEMPO sample obtained at 30 K using the spiral microresonator with the sample placed (A) directly on top of the microresonator (10 nL sample volume) and (B) within the microstructural cavity (1.6 nL). The EDFS measurements of the same samples obtained using the Bruker MD5 resonator and the same measurement time are presented for comparison yielding only the impurity signal of the sapphire ring (arrows). The EDFS of a much higher sample volume of 1 µL obtained using the MD5 resonator is presented for comparison in (A). The determined spin‐number sensitivities (spins/G/$\sqrt{\text{Hz}}$) are indicated above the measured spectra. C,D) Measured and simulated Rabi oscillations of the TEMPO sample loaded (C) directly on top of the microresonator and (D) inside the microstructure. The Rabi nutation experiments were performed at the field position of the maximum EDFS intensity by varying the duration of the first pulse and using a Hahn‐echo detection, where the π‐pulse duration was fixed to 40 ns. Small‐amplitude oscillations at longer nutation pulses in (C,D) originate from ^1^H ESEEM. Experimental parameters: droplet ‐ *Q* = 1100, τ = 400 ns, *P* = 15 mW, *t*_π_ = 40 ns; microstructure ‐ *Q* = 1900, τ = 300 ns, *P* = 6 mW, *t*_π_ = 40 ns.

The echo‐detected field‐swept (EDFS) spectrum of the ≈10 nL TEMPO droplet is presented in **Figure** **5**A revealing a typical nitroxide signal (see Figure S8, Supporting Information for the Hahn echo data). The measurement of the same droplet recorded using the Bruker MD5 resonator under the same conditions shows only the impurity signal of the sapphire ring, as the nitroxide signal is not detected (Figure 5A) due to lack of sensitivity to such a small volume sample.

We quantified the spin‐number sensitivity of both setups, defined as the minimum number of spins required to obtain the signal‐to‐noise ratio (SNR) of one. For this purpose, we also measured 1 µL of TEMPO sample using the sapphire ring resonator. In this case, we observed the nitroxide spectrum with a similar SNR compared to the measurement with the spiral microresonator despite using 100 × larger sample volume (Figure 5A). The estimation of the spin‐number sensitivity from the experimental data (see Section S5, Supporting Information for details) yields values of 2 × 10^8^ and 2 × 10^10^ spins/G/$\sqrt{\text{Hz}}$ for the spiral microresonator and the Bruker MD5 resonator, respectively. This indicates that our microresonator is significantly more sensitive than a standard EPR setup for tiny sample volumes allowing to drastically reduce the measurement time by a factor of 10,000.

Following the quantum optics description of pulsed EPR presented by Bienfait et al.,^[^ ^25^ ^]^ we derived a theoretical prediction for Hahn echo spin‐number sensitivity based on the Purcell enhancement of the spontaneous microwave photon emission in a resonator.^[^ ^50^ ^]^ combined with Dicke's model of coherent photon emission^[^ ^51^ ^]^ (see Equation (S12) and Section S5, Supporting Information for details). Our calculations yield 3.6 × 10^8^ and 1.7 × 10^10^ spins/G/$\sqrt{\text{Hz}}$ for the spiral microresonator and the Bruker MD5 resonator, respectively. The predicted sensitivities are in a good agreement with the experimentally determined sensitivities demonstrating the validity of the model.

In contrast to spin‐number sensitivity, the concentration sensitivity scales as *V* ^−1/2^ (see Equation (S13), Supporting Information) implying that microresonators generally exhibit lower concentration sensitivity compared to 3D cavities. The concentration sensitivity of our spiral microresonator with a droplet sample is 42 nM/G/$\sqrt{\text{Hz}}$, which corresponds to about 25 µM/echo for the nitroxide sample. This demonstrates that, despite a significant reduction in mode volume, our spiral microresonators still maintain acceptable concentration sensitivity.

**Figure 6:**
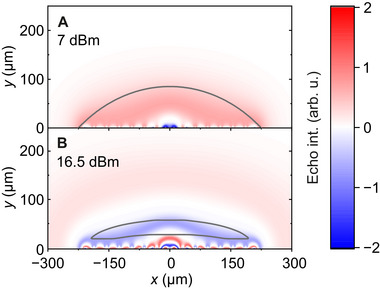
Spatial dependence of contributions to the Hahn echo signal. Spatial distribution of the Hahn echo signal under pulse powers of (A) 7 dBm and (B) 16.5 dBm obtained using COMSOL simulations. The shown power levels are optimal for the echo intensity, when using (A) a droplet or (B) a microstructural cavity. Overlaid curves show cross sections of the (A) microresonator mode volume (spherical cap, 7 nL) and (B) a microstructural cavity (1.6 nL sample volume).

Despite the substantially improved spin number sensitivity, the highly inhomogeneous *B*_1_ field of the microresonators poses challenges for pulsed EPR experiments. This is illustrated in the rapidly decaying Rabi oscillations presented in Figure 5C, showing a single dip at 40 ns with the inversion efficiency of 25% (see Figure S9, Supporting Information for additional Rabi traces). Due to the broad distribution of the *B*_1_ field, only a fraction of spins experience an exact π‐pulse (spins close to the spiral are over‐rotated, while more distant spins are only weakly perturbed). The effect is evident from the simulated contribution of different spins to the Hahn echo signal in **Figure** **6** revealing band‐like intensity distributions. As microwave power is increased, more bands are formed (see also Figure S10, Supporting Information), where the adjacent bands have opposite phases resulting in a partial cancellation of the Hahn echo signal. Based on these calculations, we simulated the measured Rabi trace for a droplet sample showing a good agreement with the experimental results (Figure 5C).

One solution to the inhomogeneity of the *B*_1_ field is to confine the spins in space to regions, where they will undergo similar rotation angles. We designed and fabricated such a microstructural cavity with 1.6 nL volume and loaded it with the 0.1 mM TEMPO sample via microfluidic channels (Figure 6B; Figure S11, Supporting Information). After determining the additional resonance frequency shift introduced by the microstructure (Figure 4B), we performed EDFS and Rabi nutation experiments, shown in Figure 5B,D. As with the droplet case, the measurement of the same sample using the Bruker MD5 resonator yielded only the impurity signal of the sapphire ring, while the EDFS obtained with the microstructure demonstrated a clear nitroxide signal with the spin‐number sensitivity of 6 × 10^6^ spins/G/$\sqrt{\text{Hz}}$. Our theoretical prediction based on Equation (S12) (Supporting Information) results in the spin‐number sensitivity of 9 × 10^7^ spins/G/$\sqrt{\text{Hz}}$ suggesting that the experimentally obtained value is better than predicted. This discrepancy is likely caused by the additional sample remaining in the microfluidic channels, which contributes to the spin signal and thus effectively increases the measured sensitivity value. Assuming the channels are fully filled, we obtain the experimental spin‐number sensitivity of 3 × 10^7^ spins/G/$\sqrt{\text{Hz}}$, which is three orders of magnitude better sensitivity compared to the Bruker MD5 resonator.

In addition to the superior spin‐number sensitivity, the microstructure also provides a significantly better spin inversion and multiple well‐resolved Rabi oscillations compared to the droplet case as indicated in Figure 5D and Figure S9 (Supporting Information). We simulated the measured Rabi trace by taking into account the shape of the microstructure (Figure 6B) yielding a satisfactory agreement with the experiment (Figure 5D). These results demonstrate that planar microresonators with appropriately adjusted sample geometry can be used to achieve high‐fidelity spin control of ordinary samples even with simple rectangular microwave pulses.

### 2.3 ESEEM, ENDOR, and DEER Spectroscopies

We tested the applicability of our spiral microresonator for commonly applied advanced pulsed EPR experiments including ESEEM, DEER, and ENDOR of different samples. Here, we employed the droplet approach instead of the microstructural cavity, which had already been exposed to the 0.1 mM TEMPO sample.

**Figure 7:**
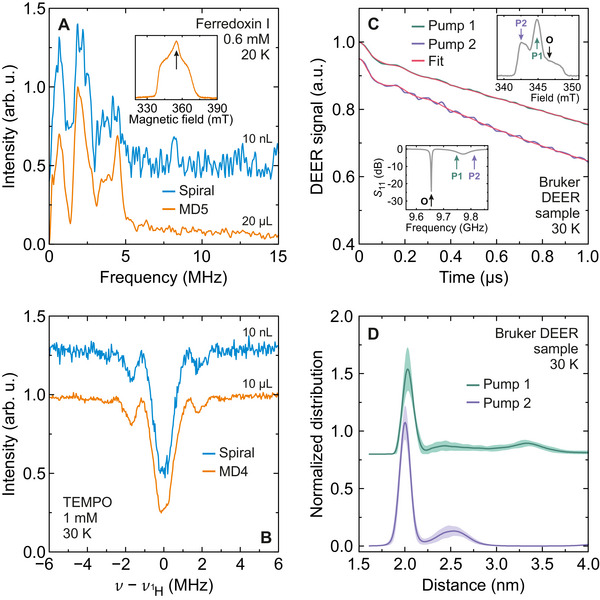
ESEEM, ENDOR, and DEER spectroscopy with spiral microresonators. A) Normalized three‐pulse ESEEM spectrum of a 0.6 mM reduced Ferredoxin I sample obtained at 20 K using the spiral microresonator (10 nL sample volume) and Bruker MD5 resonator (20 µL). Measurements were performed at the field position of the maximum EDFS intensity (*g* = 1.96) of the [2Fe‐2S]^+^ cluster signal (inset). Experimental parameters: *Q* = 1500, τ = 200, *P* = 6 mW (microresonator), *t*_π/2_ = 16 ns. B) Mims ENDOR spectrum of a 1 mM TEMPO sample obtained at 30 K using the spiral microresonator (10 nL) and Bruker MD4 resonator (10 µL). The same number of averages was used in both cases. Measurements were performed at the field position of the maximum EDFS intensity. Experimental parameters: *Q* = 1100, τ = 400 ns, *P* = 6 mW (microresonator), *t*_π/2_ = 20 ns. C) Normalized time‐domain DEER traces of a Bruker DEER sample obtained at 30 K using the spiral microresonator. Two pump positions (*P*_1_ and *P*_2_) within the mode of the MD5 resonator were explored, while the observer frequency (*O*) was tuned to the resonance frequency of the microresonator (insets). Red curves indicate best fits to the time‐domain data. The trace obtained at the pump position *P*_2_ is shifted for clarity. Experimental parameters: *Q* = 1500, *P* = 1 mW (observer), *t*_π_ = 40 ns. D) The corresponding nitroxide‐nitroxide distance distributions. The shaded regions mark the uncertainty estimate of the distance distributions.

Three‐pulse ESEEM experiments were performed at 20 K on a droplet containing 0.6 mM iron‐sulfur protein (ferredoxin I from *Thermosynecoccus elongatus*, see Experimental Section) placed on the spiral microresonator. The obtained ESEEM spectrum is presented in **Figure** **7**A revealing ^14^N signals of the protein backbone coupling to the cysteine‐coordinated [2Fe‐2S]^+^ spin.^[^ ^52^ ^]^ Measurements were performed at the field position of the maximum EDFS intensity (inset in Figure 7A), and the time‐domain ESEEM data are presented in Figure S12 (Supporting Information). For comparison, we also conducted ESEEM measurements of the same protein system using the Bruker MD5 resonator (Figure 7A). Note that here the MD5 resonator provides a higher SNR due to much higher sample volume of 20 µL compared to the typical droplet volume (10 nL) used for measurements with the spiral. Accounting for different sample volumes, the sensitivity analysis based on the frequency‐domain ESEEM spectrum reveals that the spiral microresonator has 110 × higher spin‐number sensitivity in a good agreement with the nitroxide EDFS measurements.

We also performed pulsed ENDOR spectroscopy using the spiral microresonator. Here, we employed a Bruker MD4 resonator to enable use of its ENDOR coils to generate the driving radio frequency field, with the EPR detection performed via the spiral microresonator. We used a droplet (10 nL) of 1 mM TEMPO sample placed on top of the spiral, and the obtained ^1^H Mims ENDOR spectrum is presented in Figure 7B. As above, a comparison of the SNR obtained with a 10 µL sample using the Bruker MD4 resonator shows an increase in sensitivity by factor of 450 × confirming that these spiral micoresonators are compatible with ENDOR measurements. The obtained somewhat larger sensitivity gain may indicate different coupling of the spiral to the Bruker MD4 and MD5 resonators.

DEER spectroscopy requires two microwave frequencies, typically separated by at least tens of MHz,^[^ ^1^ ^]^ posing challenges for superconducting microresonators due to their narrow resonance modes. Our setup addresses these challenges by leveraging the broad 3D cavity mode, which enhances the microwave magnetic field at the microresonator even for off‐resonance frequencies. This enhancement enabled us to easily achieve a π‐pulse duration of 40 ns, while detuned 70 MHz from the microresonator mode, with power levels only 10 dB higher (10 mW) than those required for on‐resonance operation. This configuration allowed for a wide frequency separation between the pump and observer pulses, with the pump applied close to the 3D cavity mode and the observer on the microresonator mode (see pump position *P*_1_ in the insets in Figure 7C). An even greater detuning of 130 MHz was achieved (pump position *P*_2_) for the same π‐pulse duration with 26 dB higher power (400 mW). We also explored pumping at a similar magnitude of detuning on the opposite (low‐frequency) side of the microresonator mode, where the 3D cavity does not enhance the microwave field. In this scenario, significant off‐resonance effects were observed resulting in a marked reduction of the Hahn echo signal to a practically unmeasurable level.

For DEER measurements, we used several grains of Bruker nitroxide DEER powder sample mixed with inert vacuum grease and placed on the spiral microresonator. The size of the mixed sample roughly corresponded to the mode volume of the microresonator. The DEER traces (obtained for pump positions *P*_1_ and *P*_2_) revealed oscillations (Figure 7C) corresponding to the expected nitroxide‐nitroxide distance of approximately 2 nm, as revealed by the obtained distance distributions (Figure 7D). Note that, as shown above, such a small sample amount is virtually undetectable using the MD5 cavity. These results highlight the advantage of the coupled microresonator and 3D cavity for highly sensitive DEER spectroscopy of volume‐limited samples, while supporting a large frequency separation between pump and observer pulses.

## 3 Prospects and Conclusion

Spiral YBCO microresonators coupled via standard 3D cavities are a promising, accessible, and versatile platform for highly sensitive EPR spectroscopy. The small mode volume of our microresonators can be readily used for EPR of volume‐limited systems such as single protein droplets,^[^ ^53^ ^]^ and they are also compatible with a range of sample morphologies, including frozen solutions, powders, and thin films. Single‐crystal EPR studies can also be performed by rotating the sample tube with a crystal attached to the spiral. The magnetic‐field resilience of our microresonators could also enhance ferromagnetic resonance sensitivity,^[^ ^54^, ^55^ ^]^ with YBCO providing a more robust response to local fields from adjacent magnetized samples. Functionalization of the YBCO surface (e.g., with gold)^[^ ^56^ ^]^ offers new opportunities for EPR of anchored molecules and self‐assembled monolayers containing a tiny number of spins. Spiral microresonators can be produced at a modest cost similar to other EPR consumables (such as EPR tubes) and could be used as disposable, single‐use devices, or coated with an inert material to enhance their resilience to moisture and reusability.

While the results above have targeted X‐band operation, the same approach can be extended to other frequencies such as S‐band (3 GHz) and Q‐band (35 GHz). We expect a slightly lower sensitivity improvement for the Q‐band case due to the inherently smaller volumes of Q‐band 3D cavities. As revealed by our preliminary experiments, the approach we have presented is also compatible with CW EPR experiments (see Figure S13, Supporting Information), with magnetic field modulation conveniently provided using the modulation coils commonly incorporated within the 3D resonator housing. Although YBCO spiral microresonators are limited to relatively low maximum CW powers (≈0.1 mW), their high conversion factor enables them to generate *B*_1_ fields comparable in magnitude to those used in conventional 3D cavity CW EPR.

We have shown that using commercial 3D cavity probeheads to couple to the microresonators provides a convenient method for sample loading and resonator tuning, while also enabling pump and radio frequency pulses in DEER and ENDOR spectroscopies. Nevertheless, alternative coupling methods are also possible, for example, using coplanar waveguides or other types of transmission line. Due to their small size (<1 mm), multiple microresonators could be inserted into the same EPR tube or along the same transmission line, enabling the simultaneous acquisition of multifrequency EPR, of particular relevance to systems that exhibit significant orientation selectivity.^[^ ^5^, ^15^, ^57^ ^]^ Recent advances in EPR spectrometers based on arbitrary waveform generators (AWGs)^[^ ^58^ ^]^ would be helpful in efficiently implementing such a multiplexed approach.

A general limitation of microresonators compared to 3D cavities is their poor *B*_1_ field homogeneity, however, as we have shown, this can be mitigated using microstructures for sample loading via microfluidic channels. The microstructures we employed in this work were more challenging to use and significantly more expensive than the droplet‐based approach highlighting a clear opportunity for further development of microstructures tailored to various applications in conjunction with the microresonator design. A second feature, specific to superconducting microresonators, is their elevated Q‐factor compared to fully overcoupled 3D cavities. This increases the spectrometer dead time due to microresonator ringing, which can pose challenges for certain pulsed EPR experiments. Nevertheless, we have measured Hahn echoes with spirals using a relatively short inter‐pulse delay τ of 200 ns. Resonator ringing can also be partially suppressed using shaped pulses.^[^ ^59^, ^60^ ^]^

In conclusion, spiral microresonators with 1–10 nL mode volumes, such as demonstrated here, fill an important gap between the 3D cavities commonly used in EPR and the femtolitre‐scale volume resonators^[^ ^27^, ^29^ ^]^ used to push the ultimate limits of spin number sensitivity (at the cost of spin concentration sensitivity and fabrication complexity). We have shown how such resonators, particularly when fabricated using a high‐*T*_*c*_ superconductor such as YBCO, offer a highly versatile method to enhance the sensitivity of EPR measurements. We demonstrated a variable microwave coupling of the microresonators via a 3D cavity making our approach fully compatible with conventional EPR setups. The microresonators may be operated at temperatures up to 80 K and fields up to 1 T and were validated using a suite of X‐band pulsed EPR experiments, including ESEEM, ENDOR, and DEER spectroscopies for different samples. The spin‐number sensitivity provided by our setup is two‐three orders of magnitude greater than that of typical 3D cavities, while our approach retains the reliability and user‐friendliness of conventional EPR spectroscopy, which is crucial for extending superconducting microresonators to a wide range of applications.

## 4 Experimental Section

### Microresonator Fabrication

The spiral microresonators were fabricated using a 330 nm‐thick YBCO film (M‐type) deposited by Ceraco Ceramic Coating GmbH on a 430 µm‐thick r‐cut sapphire substrate. After a cleaning step involving 5 min of sonication in acetone and then 5 min in isopropyl alcohol (IPA), the microresonator structures were patterned on the YBCO film using optical lithography. This process involved spin‐coating of S1805 positive photoresist, baked at 115° C for 60 s, followed by exposure using a Heidelberg DWL 66+ Laser Writer and development of photoresist using MF‐319. The film was subsequently ion milled for 65 min with an argon plasma in an SVS 6000 system, and the residual resist was removed by 5 min of sonication in acetone and IPA. Afterward, the substrate was diced into individual spirals using a diamond blade for cutting sapphire substrates. For protection of the microresonators during dicing, the substrate was covered with two layers of S1818 G2 photoresist, which was subsequently removed with acetone and IPA before conducting EPR experiments.

Wet etching of the YBCO film as an alternative and cheaper method was also tested to fabricate the spiral microresonators. In this case, AZ1518 photoresist was used, and the structures were etched with H_2_O:H_2_O_2_:H_3_PO_4_ (200:1:1) solution for 360 s. After etching, the photoresist was removed by rinsing in acetone. The fabricated microresonators were of similar quality as obtained using the ion milling approach. In addition, given the relatively large size of the spiral windings, the photomask lithography can also be used eliminating the need for costly laser writing equipment used in this work.

### Microstructure Fabrication

To better match the *B*_1_ field distribution (mode profile) of the microresonator with the shape and volume of the sample, microstructural cavities fabricated by LightFab GmbH from fused silica using selective laser‐induced etching were used. After fabrication, the microstructures were plasma etched for 5 min in an oxygen atmosphere to form a highly wettable surface. The liquid samples were loaded by utilizing the capillary action after placing a tiny drop (approximately 10 nL volume) of a sample on a channel connected with the microstructural cavity. Excess sample was removed using a soft tissue.

### Microresonator Characterization

The microwave scattering matrix parameter *S*_11_ of the fabricated microresonators was measured using an S5243 44 GHz Copper Mountain VNA. The resonance mode was approximated using a Fano resonance lineshape^[^ ^61^ ^]^ allowing to obtain the resonance frequency ν_0_, linewidth κ and prominence of the studied microresonators. The Q‐factor was calculated as *Q* = ν_0_/κ.

### EPR Experiments

The pulsed EPR experiments were performed using a Bruker Elexsys E580/IF‐Q X‐band EPR spectrometer equipped with a Bruker ER4118X‐MD5 microwave resonator. To enhance EPR sensitivity, an EPR cryoprobe similar in design as described in ref. [62] was employed. For pulsed ENDOR experiments, a standard unmodified Bruker EN4118X‐MD4 probehead was used. A helium‐flow CF935 cryostat was used to stabilize the sample temperature.

The EDFS spectra were recorded using the Hahn echo pulse sequence (π/2‐τ‐π‐τ‐echo), with the interpulse delay τ set to exceed the microresonator ringdown time. Rabi oscillations were measured using a three‐pulse sequence (θ‐τ′‐π/2‐τ‐π‐τ‐echo), where the duration θ of the nutation pulse was incremented. The interpulse delay τ′ was chosen to be significantly longer than the spin coherence time *T*_2_. Three‐pulse ESEEM experiments (π/2‐τ‐π/2‐τ′‐π/2‐τ‐echo) were performed by sweeping the interpulse delay τ′ with τ set to 200 ns. The pulsed ENDOR experiments were performed using the Mims ENDOR pulse sequence (π/2‐τ‐π/2‐π_*RF*_‐π/2‐τ‐echo), where the radio frequency pulse length π_*RF*_ was set to 8 µs. Depending on the experiment, two‐ or four‐step phase cycling was employed to cancel unwanted echoes. The shot repetition time was optimized depending on the sample and temperature.

The DEER experiments were performed at 30 K using the four‐pulse DEER sequence (π/2‐τ_1_‐π‐*t*_1_‐π_pump_‐(τ_1_ + τ_2_ − *t*_1_)‐π‐τ_2_‐echo).^[^ ^63^ ^]^ The DEER traces were acquired using τ_1_ = 250 ns, τ_2_ = 1.5 µs, and the initial value of *t*_1_ = 80 ns. The length of the π‐pulse was 40 ns. The traces were collected using 70 and 133 MHz frequency separations between the observer and pump pulses. An eight‐step nuclear modulation averaging with an averaging time step of 8 ns was employed. A two‐step phase cycling, an 20 ns increment of the time‐domain traces, and 3 ms shot repetition time were used. The DEER data analysis was performed using user‐independent data processing with the ComparativeDEERAnalyzer version 2.0 with DEERNet Spinach SVN Rev 5662^[^ ^64^ ^]^ and DeerLab 0.9.1.^[^ ^65^ ^]^

### Sample Details and Microresonator Stability

To benchmark the performance of the spiral microresonators for pulsed EPR and ENDOR, 4‐Amino‐TEMPO (Sigma‐Aldrich) free radical diluted to 0.1 or 1 mM in a 4:1 mixture of H_2_O:glycerol (v:v) was used. To prevent rapid sample evaporation during sample loading into the microstructure, the glycerol fraction was increased resulting in H_2_O:glycerol ratio of 3:2.

For ESEEM spectroscopy, a 0.6 mM solution of Ferredoxin I from *Thermosynechococcus elongatus* (PDB: 6JO2) was used in an aqueous tris‐HCl buffer (pH 7.5)/glycerol mixture (9:1 v/v) (see Section S6, Supporting Information for more details), which was reduced with 1 µL of an aqueous 200 mM sodium dithionite solution.

For DEER spectroscopy, a Bruker nitroxide DEER powder sample (E3005315 calibration sample) was used, which was mixed with inert Apiezon vacuum grease. To minimize any impact on sensitivity, the layer of vacuum grease covering the crystallites was significantly smaller than the mode volume of the spiral. A small amount of the obtained mixture was transferred on top of the microresonator using a toothpick. The same vacuum grease and sample loading were used to perform CW EPR spectroscopy experiments of a Bruker coal sample.

All liquid samples were deposited on top of the microresonator using a micropipette. The microresonators with the samples were carefully loaded into 4 mm quartz tubes and subsequently frozen by submerging in liquid nitrogen.

It is well known that YBCO degrades upon exposure to water leading to reduced superconducting properties or even complete failure of the film.^[^ ^66^, ^67^ ^]^ In these experiments, deterioration of spirals was likewise observed after prolonged contact with aqueous droplet samples, particularly when residues were not removed. To mitigate this effect, the spirals were cleaned with IPA after use enabling multiple reuses and long‐term storage under ambient conditions although in rare cases complete degradation of the spiral structure occurred.

### Simulation Details

The spatial distribution of the driving microwave magnetic field $B_{1}\left(r\right)$ of a resonator could be expressed as $B_{1}\left(r\right)=A_{\text{pulse}}\delta B_{1}\left(r\right)$.^[^ ^68^ ^]^ Here, $\delta B_{1}\left(r\right)$ denotes vacuum microwave magnetic field fluctuations, and a scaling factor *A*_pulse_ takes into account the amplitude of a microwave pulse. The 2D distribution of $\delta B_{1}\left(r\right)$ was calculated for the spiral microresonator cross‐section using Physics package in COMSOL Multiphysics 6.1 simulation toolbox (see Section S1, Supporting Information for more details). The spatial distribution of the spin‐resonator coupling $g_{0}\left(r\right)$ was obtained from the vacuum microwave magnetic field fluctuations as $g_{0}\left(r\right)=\gamma_{e}\delta B_{1}\left(r\right)$.

The simulated distribution of $B_{1}\left(r\right)$ was used to calculate the Hahn echo intensity of a spin system using a custom Matlab R2021b (The MathWorks Inc.) script. In these calculations, each cross‐sectional pixel of the COMSOL simulation contributes to the Hahn echo intensity as $\left|g_{0}\left(r\right)\right|\sin^{3}\left[\theta (r)\right]$,^[^ ^69^, ^70^ ^]^ where $\theta \left(r\right)=\gamma_{e}\delta \left|B_{1}\left(r\right)\right|A_{\text{pulse}}t_{\text{pulse}}$ is the flip angle, γ_*e*_ is the electron gyromagnetic ratio, and *t*_pulse_ is the pulse length. The maximum Hahn echo intensity is obtained for θ(r)=π/2. To account for the perpendicular EPR transitions measured in this work, $\left|B_{1}\left(r\right)\right|$ denotes the microwave magnetic field component perpendicular to the external magnetic field $B_{0}$. In these calculations, it was assumed that $B_{0}$ lies within the plane of the microresonator, which was a reasonable approximation, as the microresonator was typically oriented flat within the EPR tube upon insertion. The total Hahn echo amplitude was calculated by summing contributions from all pixels, weighted by their distance from the symmetry axis of the spiral cross‐section to account for the 3D spin distribution. In our simulations, *t*_pulse_ to 40 ns was set, while *A*_pulse_ was varied to find the maximum signal amplitude matching the experimental conditions.

Similarly, to simulate the Rabi nutation sequence, each contribution to the Hahn echo was multiplied by a factor of $\cos\left[\theta_{\text{Rabi}}\left(r\right)\right]$, where $\theta_{\text{Rabi}}\left(r\right)=\gamma_{e}\left|B_{1}\left(r\right)\right|A_{\text{pulse}}t_{\text{Rabi}}$, and *t*_Rabi_ is the duration of the nutation pulse. By varying *t*_Rabi_, this approach allowed to reproduce the experimentally observed Rabi oscillations.

The microwave coupling of the spiral microresonator was simulated using a Frequency Domain Solver in CST Studio Suite package. An adaptive tetrahedral mesh refinement was employed with the minimum mesh cell size adjusted to accurately sample the microresonator geometry. The microresonators were modeled as perfect electrical conductors of 0.3 µm thickness placed on a sapphire substrate. The microresonator properties and coupling were studied by either placing it adjacent to a coplanar waveguide transmission line or by inserting it into a sapphire ring resonator corresponding to a Bruker MD5 resonator assembly (see Figure S1, Supporting Information). For simulation with the sample, the dielectric permittivity of ε = 3.2 matching that of frozen water, was used.

## Conflict of Interest

The authors declare no conflict of interest.

## Author Contributions

G.U. and M.Š. contributed equally to this work. Conceptualization is performed by G.U., M.Š., O.W.K., M.M.R., and J.J.L.M.; Data curationis performed by G.U., M.Š., B.G., I.P., and P.V.; Formal analysis is provided by G.U., M.Š., B.G., I.P., and P.V.; Funding acquisition is provided by M.Š., V.K., J.B., M.M.R., and J.J.L.M.; Investigation is done by G.U., M.Š., B.G., O.W.K., I.P., P.H., A.V.R.T., J.‐B.V., A.C., J.A., V.K., M.M.R., and J.J.L.M.; Methodology is provided by G.U., M.Š., O.W.K., V.K., and J.J.L.M.; Project administration is provided by M.Š., J.B., M.M.R., and J.J.L.M.; Resources are provided by M.Š., O.W.K., B.G., P.H., A.V.R.T., J.‐B.V., A.C., G.A.J., M.K., M.T., P.G., M.M.R., and J.J.L.M.; Software is provided by G.U., M.Š., I.P., and P.V.; Supervision is done under M.Š., J.B., M.M.R., and J.J.L.M.; Validation is provided by G.U., M.Š., B.G., M.M.R., and J.J.L.M.; Visualization is performed by G.U., and M.Š.; Writing—original draft is provided by G.U. and M.Š.; Writing‐review and editing is completed by all authors. All authors have read and agreed to the published version of the manuscript.

## Supporting information

Supporting Information

## Data Availability

The data that support the findings of this study are openly available in MIDAS at https://doi.org/10.18279/MIDAS.SPECTR.203438.
