# Supplementary material for: Versatile High‐Sensitivity EPR Using Superconducting Spiral Microresonators
Source: Small Methods. 2025 Nov 12;10(6):e01451. doi: 10.1002/smtd.202501451 (PMC13010201; doi:10.1002/smtd.202501451)
Supplement: Supplementary file 1 — Supporting Information [file SMTD-10-e01451-s001.pdf]

Supplementary Information for Versatile high-sensitivity EPR using superconducting spiral microresonators

Gediminas Usevičius, Mantas Šimėnas,* Blaise L. Geoghegan, Oscar W. Kennedy, Ignas Pocius, Patrick Hogan, Ana Villanueva Ruiz de Temino, Jean-Baptiste Verstraete, G. Antilen Jacob, Paulina Verbaitytė, Angeliki Chatziathanasiou, Mindaugas Kamarauskas, Marius Treideris, Paulius Gečys, Joseph Alexander, Vidmantas Kalendra, Jūras Banys, Maxie M. Roessler, and John J.L. Morton*

E-mail: mantas.simenas@ff.vu.lt; jjl.morton@ucl.ac.uk

1 Resonator impedance and microwave magnetic field

We calculated the current density profile $\delta J(x)$ of a superconducting YBCO wire having the same cross section as a single winding of the spiral microresonator. The current density was assumed to be same along the wire. For the calculations of $\delta J(x)$, we used the following expression:[1,2]

$$\delta J(x) = \begin{cases} \delta J(0)[1-(2x/w)^2]^{-1/2} & \text{for } |x| \leq |\frac{1}{2}w - \lambda^2/(2b)| \\ \delta J\left(\frac{1}{2}w\right)\exp[-(\frac{1}{2}w-|x|)b/\lambda^2] & \text{for } |\frac{1}{2}w - \lambda^2/(2b)| < |x| < \frac{1}{2}w \\ (1.165/\lambda)(wb)^{1/2}\delta J(0) & \text{for } x = \frac{1}{2}w. \end{cases} \tag{S1}$$

Here, w, b and λ denote the width, thickness and magnetic field penetration depth of the YBCO wire, respectively. In our calculations, we take $w = 20$ µm and $b = 330$ nm. The value of the normalization constant $\delta J(0)$ was determined using the following expression: $\omega_0\sqrt{\hbar/2Z_0} = b\int_{-w/2}^{w/2}\delta J(x)dx$. The impedance of the microresonator $Z_0 = \omega_0 L$ was estimated to be 426 Ω, where the geometric inductance L of the spiral was calculated using the model reported in Refs. 3, 4. Here, $\omega_0 = 2\pi\nu_0$ is experimentally determined angular resonance frequency of the spiral microresonator. In our calculations, we considered a spiral geometry resonating at $\nu_0 = 9.5$ GHz. The penetration depth λ of the used YBCO film is 260 nm at 77 K, as specified by the supplier (Ceraco Ceramic Coating GmbH). We used a power law expression reported in Ref. 5 to obtain the values of λ at lower temperatures.

The obtained superconducting current density distribution $\delta J(x)$ was used to calculate the vacuum microwave magnetic field fluctuations $\delta \boldsymbol{B}_1(\boldsymbol{r})$ of a spiral microresonator. For this purpose, we employed the COMSOL Multiphysics 6.1 simulation toolbox, where a plane containing a cross section of a spiral microresonator was simulated. An extra-fine mesh was applied during calculations to capture the sharp increase in the superconducting current density with greater accuracy. Note that the cross-section representation of the spiral is a simplification, as it neglects the effects arising from the turning of the spiral windings. In

our simulations, we also ignore the microwave field distribution due to the open ends of the spiral. The microwave magnetic field $\boldsymbol{B}_1(\boldsymbol{r})$ was obtained by scaling $\delta\boldsymbol{B}_1(\boldsymbol{r})$ based on the amplitude of the microwave excitation. The spin-resonator coupling constant $\boldsymbol{g}_0(\boldsymbol{r})$ was calculated as $\boldsymbol{g}_0(\boldsymbol{r}) = \gamma_e \delta\boldsymbol{B}_1(\boldsymbol{r})$, where γ_e is the electron gyromagnetic ratio.

2 Coupling of spiral microresonators

The microwave coupling of the spiral microresonator placed within the 3D sapphire ring resonator was studied using CST simulations. Simulation geometry is presented in Fig. S1. Our simulations indicate that the coupling depends on the orientation of the spiral within the sapphire ring. The strongest coupling is achieved, if the spiral plane is perpendicular to the microwave magnetic field $\boldsymbol{B}_1$ of the 3D resonator (Fig. S2). Thus, depending on the resonance frequency and orientation of the microresoantor, it can couple via either the $\mathrm{HE}_{11\delta}$ ($\boldsymbol{B}_1$ field perpendicular to the symmetry axis) or $\mathrm{TE}_{01\delta}$ ($\boldsymbol{B}_1$ along the symmetry axis) mode of the sapphire ring. The latter mode is dominant in our study, as typical orientation of the microresonator in the EPR tube is close to horizontal.

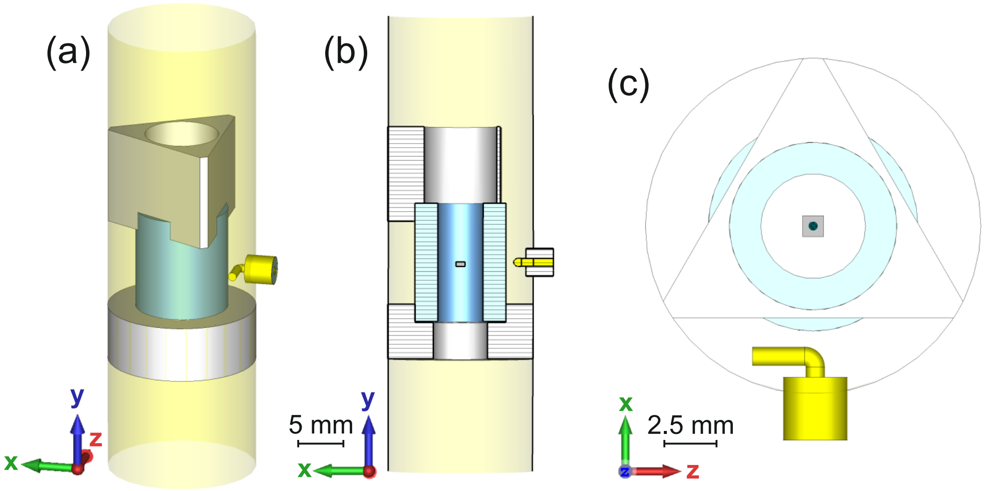

Figure S1: (a) Geometry of the Bruker MD5 sapphire ring resonator assembly containing a perfectly conducting spiral microresonator used in the CST simulations. (b) Cut in the xy-plane, and (c) view along the y-axis. The metal shield is made transparent for clarity.

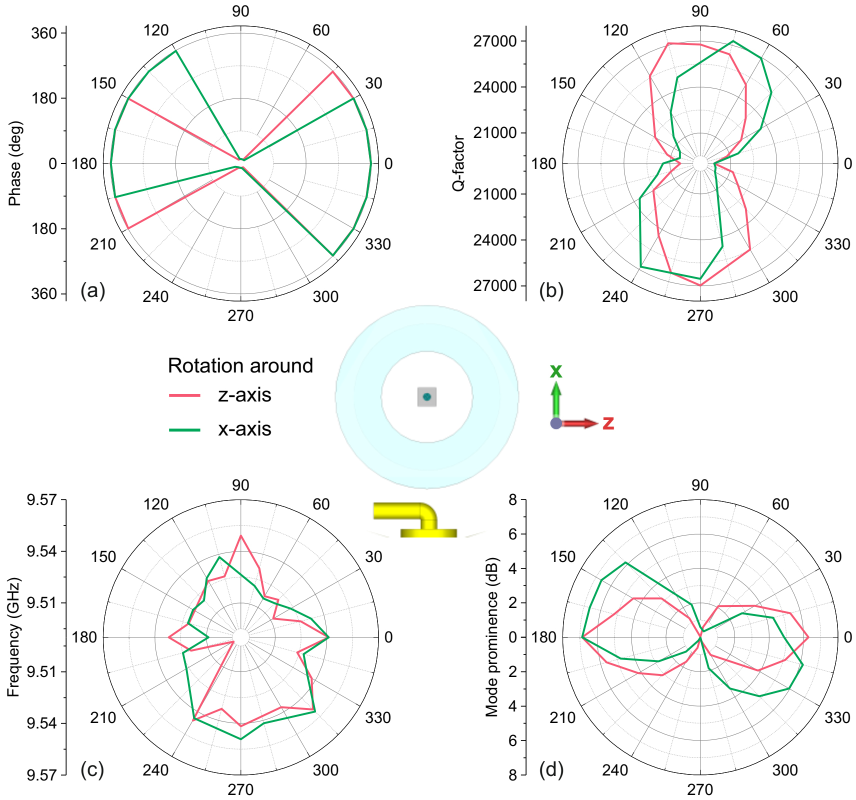

Figure S2: CST simulation of (a) phase, (b) Q-factor, (c) frequency and (d) prominence (mode depth) of a spiral microresonator resonant at about 9.5 GHz, when it is rotated around two orthogonal directions (inset) in the center of the Bruker MD5 sapphire ring resonator assembly. The overcoupling is achieved, when the normal vector of the resonator plane is pointing approximately along the symmetry axis of the sapphire ring, which coincides with its $\boldsymbol{B}_1$ field direction of the $TE_{01\delta}$ mode.

3 Characterization of spiral microresonators

In total, we characterized over 120 spiral microresonators of different resonance frequencies ranging from 8.5 to 12.5 GHz using VNA measurements (see Fig. S3). The microresonators were obtained from several independent fabrication batches. The obtained resonance frequency vs. the total spiral length is presented in Fig. S3a together with the CST simulation results. The microresonator Q-factor vs. resonance frequency is presented in Fig. S3b revealing a broad variation of the Q-factor due to different intrinsic and coupling losses. The frequency dependence of the microresonator prominence and phase are presented in Fig. S3c,d showing overcoupled regimes for frequencies that are close to the resonances of the sapphire ring. The CST simulations show a qualitative agreement with the experimental data, except for the Q-factor, where significantly higher simulated values arise due to the exclusion of intrinsic microwave losses of the microresonator.

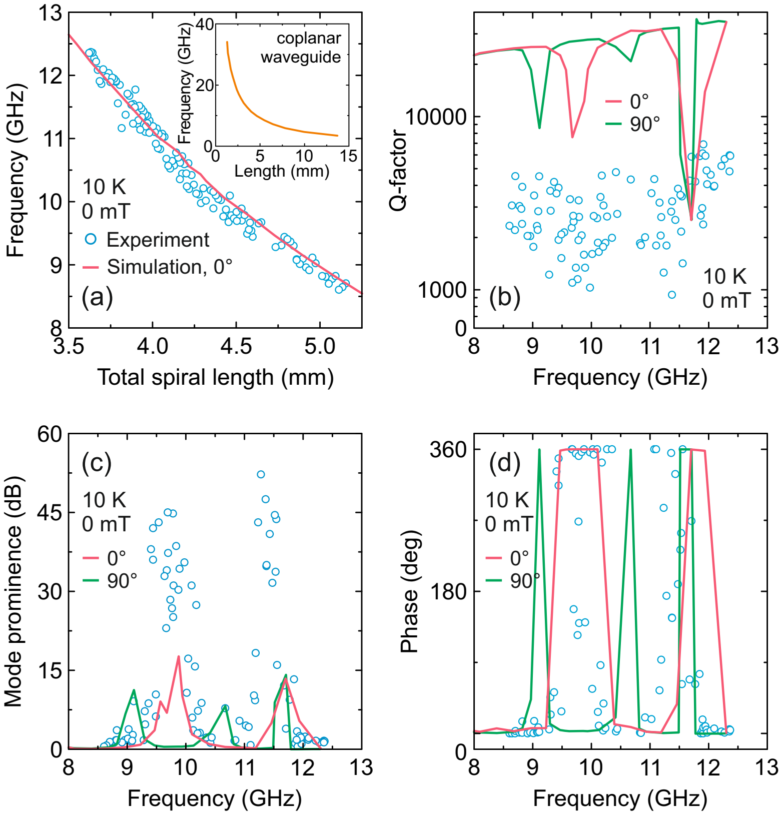

Figure S3: (a) Resonance frequency dependence on spiral length, as measured by VNA for 122 individual microresonators. Distribution of the resonant mode (b) Q-factor, (c) prominence and (d) phase vs. resonance frequency. Measurements performed at 10 K and 0 mT. The solid curves indicate CST simulations obtained using perfectly conducting microresonators placed inside a Bruker MD5 resonator at 0° (coupling via the $TE_{01\delta}$) and 90° (coupling via the $HE_{11\delta}$) angle with respect to the symmetry axis of the sapphire ring. The inset in (a) shows simulation obtained for microresonators coupled to a broadband coplanar waveguide geometry, which allowed us to simulate a much broader frequency range.

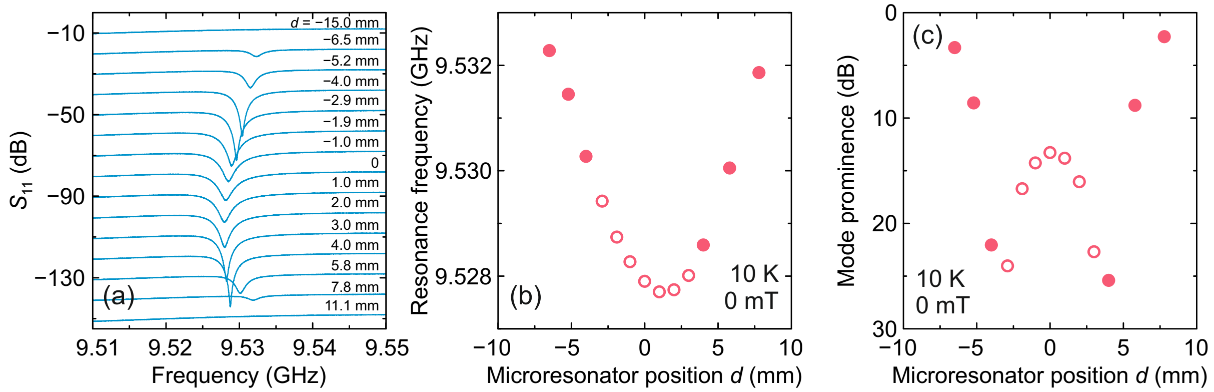

Figure S4: Stacked S_{11} VNA traces of resonant mode obtained at different distances from the center of a Bruker MD5 sapphire ring cavity. Position dependence of the (b) frequency and (c) prominence of the microresonator mode. The open points correspond to the overcoupled microresonator. Measurements performed at 10 K and 0 mT.

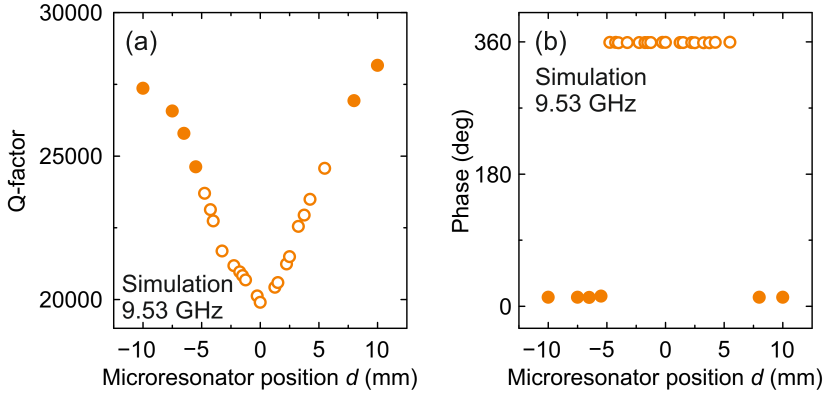

Figure S5: CST simulation of the (a) Q-factor and (c) phase of a spiral microresonator resonant at 9.53 GHz obtained at different distances from the center of a Bruker MD5 sapphire ring cavity. The open points correspond to the overcoupled microresonator mode, as evident from the change of the microwave phase by 360°.

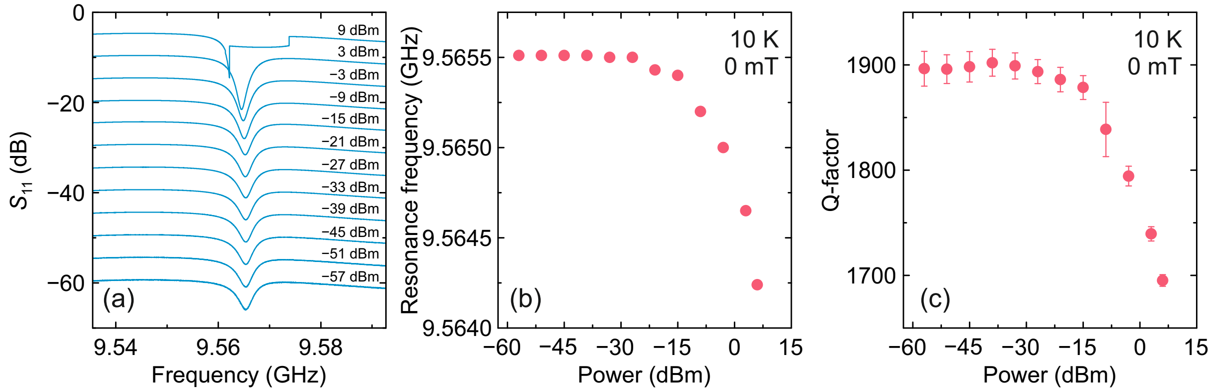

Figure S6: (a) Stacked S_{11} VNA traces of resonant mode obtained at different microwave power levels for a selected microresonator. Power dependence of the microresonator (b) resonance frequency and (c) Q-factor. Measurements performed at 10 K and 0 mT.

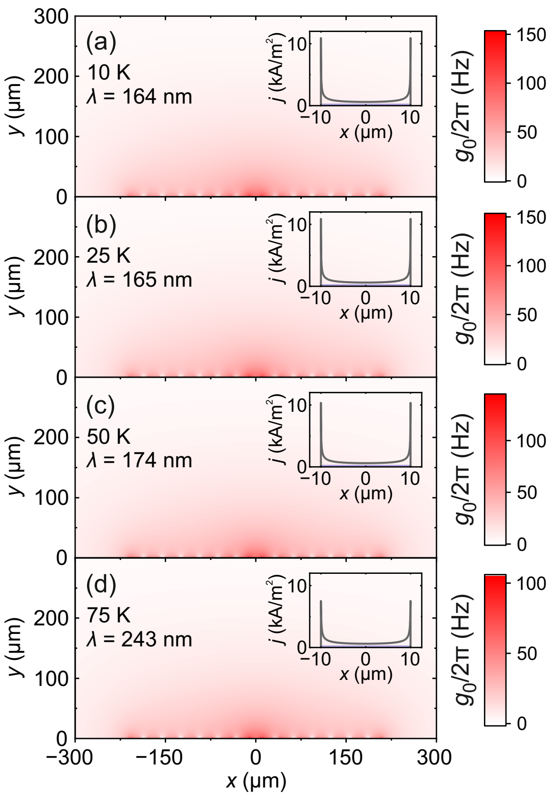

Figure S7: Spatial distribution of the calculated spin-resonator coupling strength g_0 for $g = 2$ spin species obtained at (a) 10, (b) 25, (c) 50 and (d) 75 K using COMSOL simulations. The current profiles used to obtain the g_0 distributions are presented in the insets.

4 Additional EPR data and simulations

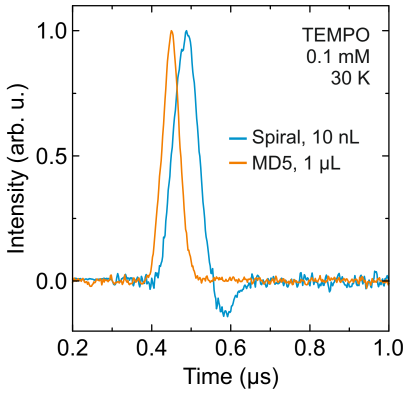

Figure S8: Hahn echo of a 0.1 mM TEMPO sample obtained at 30 K using the spiral microresonator (10 nL sample volume) and Bruker MD5 resonator (1 μL sample volume). An identical number of averages was accumulated for both measurements. The echo obtained with the microresonator shows a slight distortion due to a relatively high Q-factor ($Q = 1100$).

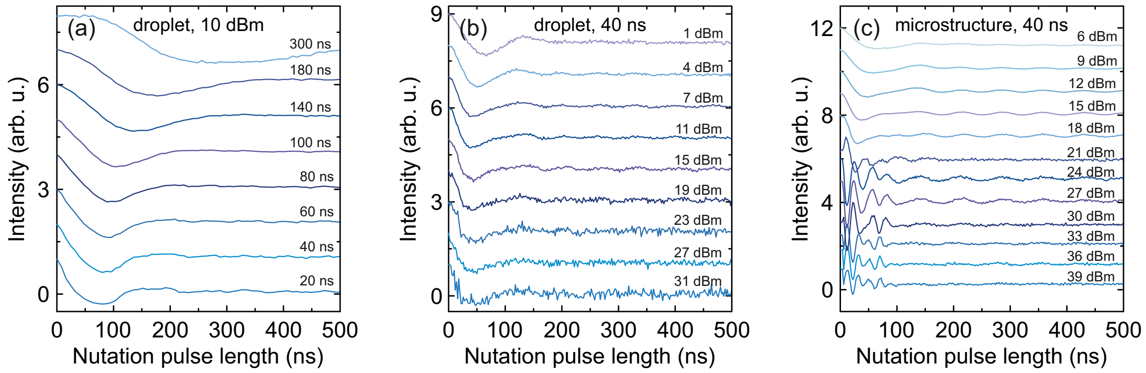

Figure S9: Stacked normalized Rabi traces of the 0.1 mM TEMPO sample placed (a,b) on top of the microresonator as a droplet and (c) within a microstructural cavity. Measurements in (a) were performed by sweeping the duration of the π-pulse in the Hahn echo detection sequence, while keeping the pulse power constant at 10 dBm. The pulse power was varied in (b,c), where the π-pulse duration was fixed to 40 ns. Due to the finite base thickness of the microstructure, good inversion was achieved using higher power levels compared to the droplet case.

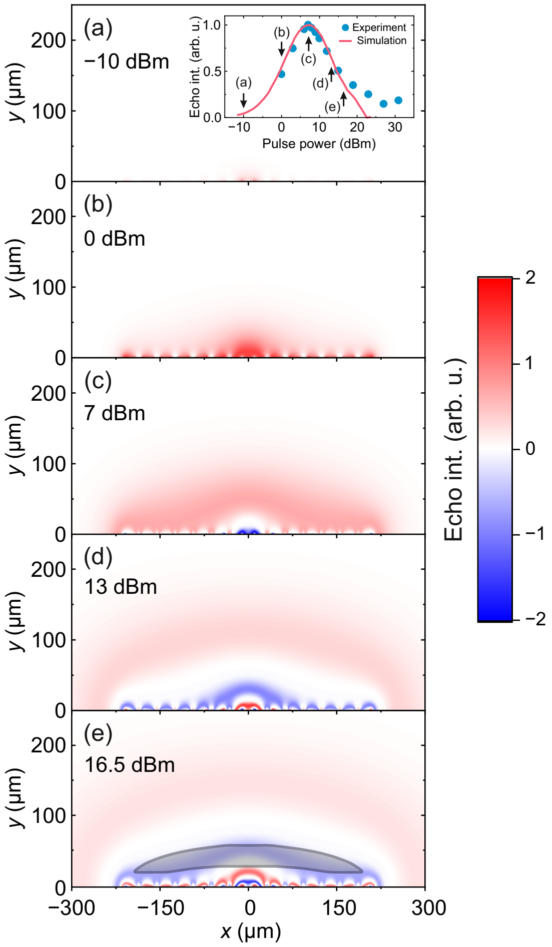

Figure S10: Spatial distribution of the Hahn echo signal contribution for different pulse power levels obtained using COMSOL simulations. Inset in (a) shows measured and simulated echo intensity vs. pulse power, which was used to calibrate power level in COMSOL simulations. In (e), the cross section of the microstructural cavity is overlaid on the intensity map.

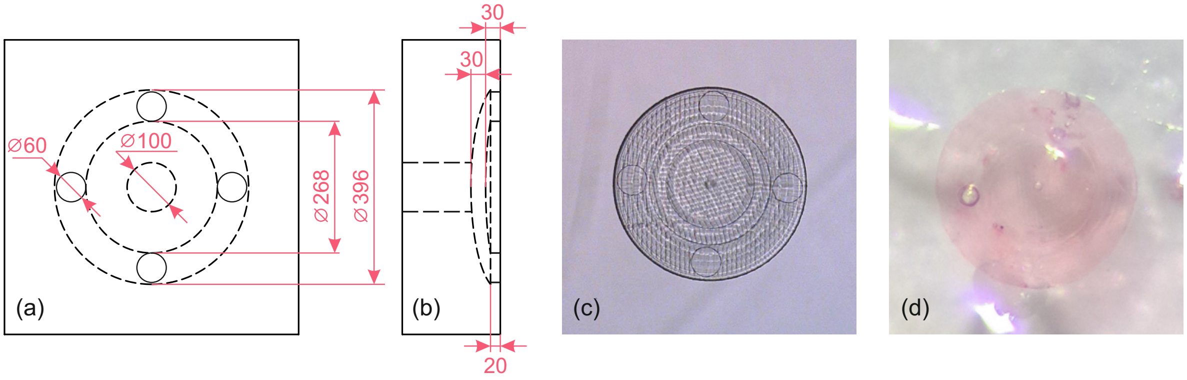

Figure S11: (a) Top and (b) side views of a schematic of the microstructural cavity with integrated channels for sample loading. All dimensions are provided in µm. A photograph of the fabricated (c) empty and (d) loaded microstrucure in fused silica. The nitroxide sample in (d) was dyed using red food dye.

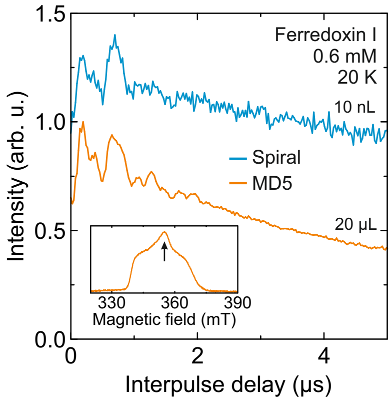

Figure S12: Three-pulse ESEEM time-domain data of a 0.6 mM Ferredoxin I sample obtained at 20 K using the spiral microresonator (10 nL sample volume) and Bruker MD5 resonator (20 μL). The trace obtained with the microresonator is shifted by 0.4 for clarity. The trace obtained with the microresonator has 50× more averages (2 vs. 100). Measurements were performed at the field position of the maximum EDFS intensity (g = 1.96, inset). Experimental parameters: τ = 200 ns, P = 6 mW (microresonator), $t_{\pi/2}$ = 16 ns.

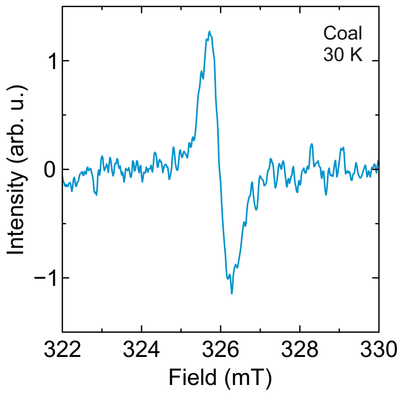

Figure S13: CW EPR spectrum of a tiny speck of a Bruker coal sample obtained at 30 K and 0.1 nW power using the spiral microresonator (Q = 2100). To perform this experiment, a modulation field of 100 kHz frequency and 3 G amplitude created by the modulation coils of the Bruker MD5 probehead was used. The same amount of samples is practically undetectable using a 3D cavity EPR.

5 Calculation of spin-number sensitivity

The spin-number sensitivity S, defined as the minimum number of spins required to obtain the SNR of one, was calculated from the experimental EDFS data using the following expression:

$$S = \frac{N}{\gamma_S} \frac{\sqrt{SRT}}{\frac{SNR}{\sqrt{a}}}. \tag{S2}$$

Here, the number of measured spins N per spin linewidth γ_S in gauss is scaled by the voltage SNR per single average $\frac{SNR}{\sqrt{a}}$. To take into account the measurement time, the resulting spin-number sensitivity is multiplied by $\sqrt{SRT}$, where SRT is the shot repetition time of our experiment.

For comparison with the experimentally obtained results, we derived a theoretical expression for the spin-number sensitivity S. Our approach is similar to the derivation presented in Ref. 2. The derivation begins by considering the spontaneous emission rate of microwave photons A_ω, which is expressed as

$$A_\omega = \frac{\mu_0 \hbar \gamma_e^2 \omega^3}{6\pi c^3}, \tag{S3}$$

where μ_0 is the vacuum permeability, $\hbar$ is the reduced Planck constant, ω is the photon angular frequency, and c is the speed of light.

In a resonant cavity, the emission rate is significantly enhanced by the Purcell factor F_p given by[6]

$$F_p = \frac{3Q\lambda^3}{4\pi^2 V}, \tag{S4}$$

where V is the cavity mode volume, Q is the cavity quality factor, and λ is the photon wavelength. The microwave photon emission rate is then expressed as

$$\Gamma_\omega = A_\omega F_p = \mu_0 \hbar \gamma_e^2 \frac{Q}{V}. \tag{S5}$$

When N coherent spins contribute to the echo, the emission rate is further enhanced as[7]

$$\Gamma_\omega = \mu_0 \hbar \gamma_e^2 \frac{Q}{V} \left(\frac{pN}{2}\right)^2, \tag{S6}$$

where p represents the spin polarization, and pN is the number of polarized spins.

The total number of photons emitted during a single echo of duration T_E is given by

$$N_E = \mu_0 \hbar \gamma_e^2 \frac{Q}{V} \left(\frac{pN}{2}\right)^2 T_E, \tag{S7}$$

and the voltage SNR can be calculated as

$$SNR = \sqrt{\frac{N_E}{n}} = \frac{pN}{2} \gamma_e \sqrt{\frac{\mu_0 \hbar Q T_E}{nV}}, \tag{S8}$$

where n is number of noise photons.

The minimum number of spins required to achieve $SNR = 1$ is given by

$$N_{min} = \frac{2}{p\gamma_e} \sqrt{\frac{nV}{\mu_0 \hbar Q T_E}}. \tag{S9}$$

For the case where the spin linewidth γ_S exceeds the resonator linewidth κ (the case in our experiments), the echo duration can be approximated as $T_E = 1/\kappa$. Additionally, to account for the spins physically located within the resonator mode volume, N_{min} is scaled by a factor of γ_S/κ.

Then the minimum number of spins can be expressed as

$$N_{min} = \frac{2\gamma_S}{p\gamma_e} \sqrt{\frac{nV}{\mu_0 \hbar \omega}}, \tag{S10}$$

and the spin-number sensitivity S, in units of spins/gauss/$\sqrt{\text{Hz}}$, is

$$S = \frac{2}{p} \sqrt{\frac{nV}{\mu_0 \hbar \omega}} \sqrt{SRT}. \tag{S11}$$

Here, the frequency-field conversion factor γ_e for the spin linewidth has been taken into account. Note that in this case the sensitivity is independent of the resonator Q-factor.

To account for the band cancellation effect caused by the inhomogeneous spatial distribution of the B_1 field, we introduce an additional factor η resulting in

$$S = \frac{1}{\eta}\frac{2}{p}\sqrt{\frac{nV}{\mu_0 \hbar\omega}}\sqrt{SRT}. \tag{S12}$$

Here, η corresponds to the inversion fraction obtained from the Rabi nutation experiments. For the droplet case, $\eta = 0.25$ (see Fig. 5C), while $\eta = 1$ for the homogeneous field distributions achieved with the 3D cavity or for sample confined in a microstructure.

To obtain the spin-concentration sensitivity, the spin-number sensitivity is divided by the mode volume resulting in

$$S/V = \frac{1}{\eta}\frac{2}{p}\sqrt{\frac{n}{\mu_0 \hbar\omega V}}\sqrt{SRT}. \tag{S13}$$

6 Purification of Ferredoxin

Ferredoxin I was expressed in BL21 *E. coli* cells containing *T. elongatus* ferredoxin (tsl1009) gene (see below) in a pET-24(+) vector. Cells were broken at 25 KPSI in resuspension buffer (50 mM Tris-HCl pH 7.5, 200 mM NaCl, 2.7 mM EDTA, 0.1% (v/v) β-mercaptoethanol and 0.1 mM Pefabloc). To remove bulky and hydrophobic contaminants, the supernatant was saturated with $(NH_4)_2SO_4$ and applied to a size exclusion (Sephadex G100) and a hydrophobic interaction column (Phenyl Sepharose, gradient 0.1-1 M NaCl in elution buffer containing 50 mM Tris-HCl pH 7.5, 100 mM NaCl, 60% saturating concentration $(NH_4)_2SO_4$). A HiPrep™ 26/10 desalting column in dialysis buffer (50 mM Tris-HCl pH 7.5, 100 mM NaCl, 2.7 mM EDTA) was used to remove $(NH_4)_2SO_4$ excess. Finally, ion exchange (0.07-1 M NaCl gradient in Q Sepharose column) and size exclusion chromatography (HiLoad 26/60 Superdex 75pg in SEC buffer (20 mM MES pH 6.5, 150 mM NaCl)) further purified ferredoxin to a final concentration of 0.6 mM.

Ferredoxin (tsl1009) gene sequence:

ATGGCAACCTACAAAGTAACGCTAGTGCGTCCTGATGGAAGCGAAACAACA
ATTGACGTGCCCGAAGATGAGTACATTCTGGATGTGGCCGAAGAGCAAGGCCT
AGACTTGCCCTTCTCCTGCCGTGCTGGTGCTTGCTCCACCTGTGCCGGTAAGC
TCCTGGAAGGAGAAGTGGATCAGTCGGATCAGTCCTTCTTGGATGATGACCAA
ATTGAGAAGGGCTTTGTGCTTACCTGTGTAGCCTATCCCCGTTCTGACTGCAA
AATCCTCACCAACCAAGAGGAAGAGCTTTACTAA

References

(1) Van Duzer, T.; Turner, C. *Principles of Superconductive Devices and Circuits*; Prentice Hall, 1999.

(2) Bienfait, A.; Pla, J. J.; Kubo, Y.; Stern, M.; Zhou, X.; Lo, C. C.; Weis, C. D.; Schenkel, T.; Thewalt, M. L. W.; Vion, D. et al. Reaching the quantum limit of sensitivity in electron spin resonance. *Nat. Nanotechnol.* **2016**, *11*, 253–257.

(3) Mohan, S.; del Mar Hershenson, M.; Boyd, S.; Lee, T. Simple accurate expressions for planar spiral inductances. *IEEE J. Solid-State Circuits* **1999**, *34*, 1419–1424.

(4) Medahinne, M.; Kandel, Y. P.; Magar, S. T.; Champion, E.; Nichol, J. M.; Blok, M. S. Magnetic-field-tolerant superconducting spiral resonators for circuit quantum electrodynamics. *Phys. Rev. Appl.* **2025**, *23*, 014070.

(5) Harshman, D. R.; Schneemeyer, L. F.; Waszczak, J. V.; Aeppli, G.; Cava, R. J.; Batlogg, B.; Rupp, L. W.; Ansaldo, E. J.; Williams, D. L. Magnetic penetration depth in single-crystal $YBa_2Cu_3O_{7-\delta}$. *Phys. Rev. B* **1989**, *39*, 851–854.

(6) Purcell, E. M. Spontaneous emission probabilities at radio frequencies. *Phys. Rev.* **1946**, *69*, 681.

(7) Dicke, R. H. Coherence in Spontaneous Radiation Processes. *Phys. Rev.* **1954**, *93*, 99–110.
